# A Rare Manifestation of CNS Leukemia: A Case Report

**DOI:** 10.1155/crh/7730665

**Published:** 2025-12-06

**Authors:** Arit Ntekim, Areeba Nayyer, Stephanie Rosales

**Affiliations:** ^1^ Department of Medicine, Englewood Health, 350 Engle St, Englewood, 07631, New Jersey, USA

**Keywords:** acute myeloid leukemia, central nervous system leukemia, intrathecal methotrexate

## Abstract

Central nervous system (CNS) involvement in acute myeloid leukemia (AML) is uncommon, reported in < 3% of patients, and confers poor prognosis. We present a 71‐year‐old Korean woman with prior myeloid sarcoma who progressed to AML and later developed isolated CNS leukemia. Her course included pancytopenia, extramedullary skin lesions, hyperleukocytosis, transfusion‐dependent anemia, and elevated LDH. Neurologic decline revealed dural lesions on imaging; cerebrospinal fluid flow cytometry confirmed CNS disease despite negative cytology. She responded to intrathecal methotrexate and high‐dose cytarabine, underscoring the need for CNS‐directed therapy. Myeloid sarcoma precedes AML in 2%–8% of cases, yet CNS relapse remains rare. Diagnostic challenges arise from nonspecific neuroimaging and overlap with infectious or inflammatory etiologies, highlighting the role of flow cytometry and molecular studies. Median survival after CNS relapse is reported at 3–6 months. This case also illustrates how language barriers may delay diagnosis and complicate management, emphasizing the need for accessible care frameworks.

## 1. Introduction

Central nervous system (CNS) involvement in leukemia presents a significant challenge in the management of hematological malignancies. This case report describes a patient initially diagnosed with myeloid sarcoma, who subsequently developed acute myeloid leukemia (AML) and CNS leukemia. Myeloid sarcoma, also known as chloroma or extramedullary myeloid tumor, is a rare manifestation of myeloid neoplasms that can occur in various anatomical sites [1]. It may precede or coincide with AML, myelodysplastic syndromes, or myeloproliferative neoplasms [1, 2]. The progression from myeloid sarcoma to AML and the subsequent development of CNS leukemia highlight the complex nature of these hematological disorders and the importance of vigilant monitoring and management strategies.

Myeloid sarcoma is reported in 2%–8% of patients with AML, either as single or multifocal tumors [1, 2]. In approximately a quarter of cases, it may predate AML by months or years [1, 2]. The incidence of CNS involvement in AML ranges from 0.6% to 3% in adults, though some authors argue that subclinical cases are likely underestimated [3–5]. The diagnosis and treatment of myeloid sarcoma, particularly when it progresses to AML with CNS involvement, require a multidisciplinary approach and careful therapeutic planning.

This case report aims to contribute to the limited literature on the progression from myeloid sarcoma to AML and the subsequent development of CNS leukemia. By presenting the clinical course, diagnostic challenges, and treatment decisions in this case, we hope to provide insights to inform future management of similar patients.

## 2. Case

The patient is a 71‐year‐old Korean woman with a past medical history of myeloid sarcoma that was diagnosed via biopsy of extramedullary hematopoiesis skin lesions. Her hematologist believed that her pancytopenia was due to chronic myelomonocytic leukemia (CMML). A CBC from 8 months prior to presentation to our facility indicated a hemoglobin of 8.8 g/DL, white cell count of 18 K/UL, and platelets of 90 K/UL. The patient was not on any active chemotherapy treatment by choice. She had a recent history of COVID‐19 infection three months before the admission. She presented to the ED with worsening fatigue, weakness, and decreased oral intake over the past week. The family reported intermittent low‐grade fevers at home; however, she spiked to a fever with a maximum temperature of 102°F a few days before coming to ED. The patient denied any chest pain, cough, rhinorrhea, or sore throat, and there were no sick contacts. The patient also denied nausea, vomiting, or diarrhea. She reported intermittent left upper abdominal pain that worsened with eating and also had constipation with the last bowel movement on the day of presentation, which was nonbloody. She also endorsed mild postvoid pain; however, there was no change in urine color or smell. The patient also had diffuse arthralgias. Of note, most of the history was obtained from family members and previous records, as the patient’s primary language is Korean and she had a limited understanding of her condition.

The patient initially presented to the emergency department (ED) afebrile and hemodynamically stable. Laboratory evaluation revealed severe pancytopenia with the hemoglobin of 6.2 g/dL, hematocrit of 17.5%, and white blood cell count of 0.17 × 10^9^/L. The mean corpuscular volume was 87 fL. No blasts or immature granulocytes were identified on peripheral smear. Workup for tumor lysis syndrome was unremarkable, with the uric acid of 1.6 mg/dL. Additional ED laboratories were notable for sodium of 127 mmol/L, potassium of 5.9 mmol/L (hemolyzed sample), lactic acid of 3.3 mmol/L, procalcitonin of 2.2 ng/mL, and lactate dehydrogenase of 3425 U/L. Urinalysis was noninfectious. A chest radiograph demonstrated bilateral haziness without consolidation. Most notable on physical examination was the respiratory exam, which showed mildly increased work of breathing and bibasilar crackles on auscultation, more pronounced at the lung bases. No wheezes or rhonchi were noted, and air entry was good throughout. On neurologic examination, the patient was oriented to person but disoriented to time. She followed simple commands, with cranial nerves II–XII grossly intact. No focal motor or sensory deficits were observed. Gait was not directly assessed at the bedside; however, family reported that she had been experiencing difficulty walking and an unsteady gait for several months prior to admission, which they had attributed to aging.

The patient was admitted and empirically started on broad‐spectrum antibiotics for suspected pneumonia. Her hemoglobin nadired at 6.2 g/dL, requiring transfusion of two units of packed red blood cells, after which her levels stabilized. Despite antibiotics, she developed recurrent fevers up to 103°F (39.4°C). Blood, urine, and sputum cultures remained negative, raising concern for malignancy‐related fevers.

**Figure 1:**
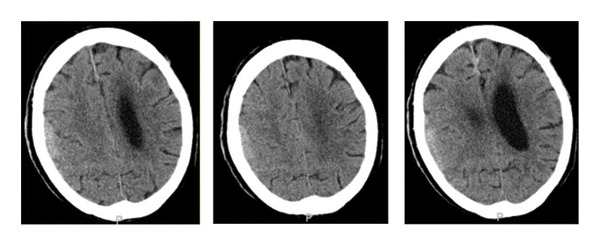
Axial noncontrast CT head images at the level of the lateral ventricles and centrum semiovale. CT demonstrates subdural hematomas along the right cerebral convexity and anterior falx cerebri, chronic infarct involving the left basal ganglia, chronic ischemic change/gliosis in the left frontal periventricular white matter, and ex vacuo dilatation of the frontal horn.

**Figure 2:**
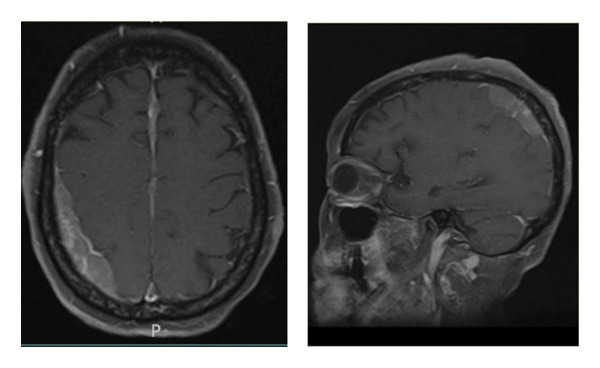
Axial and sagittal postcontrast T1‐weighted MRI brain obtained shortly after initial CT head concerning for subdural hematoma. MRI demonstrates dural thickening and enhancement along the bilateral cerebral convexities and interhemispheric falx, chronic microvascular ischemic changes, and chronic left basal ganglia infarction. There is stable abnormal decreased T1 marrow signal with heterogeneous enhancement, findings highly suggestive of an infiltrative neoplastic process rather than subdural hematoma.

Approximately a week after admission, the patient developed waxing and waning mentation. CT imaging of the head showed a subdural hematoma along the right cerebral convexity and anterior falx without acute territorial infarction (Figure1). A chronic infarct involving the left basal ganglia and periventricular ischemic gliosis was also noted. Subsequent MRI demonstrated bilateral dural‐based thickening along the cerebral convexities and falx, concerning for neoplastic or metastatic disease, including lymphoma or CNS leukemia; subdural empyema and meningitis were also considered (Figure 2).

On November 13, 2024, peripheral blood molecular studies showed no blasts on flow cytometry, though there were abnormalities including diminished granulocyte CD10/CD13/CD14/CD16 expression, aberrant CD56 coexpression, hypogranularity, and a small atypical B‐cell population comprising 2.3% of lymphocytes. Cytogenetics and FISH were negative for AML and MDS, while next‐generation sequencing identified pathogenic variants in ASXL1 Exon 12 (40.9%), SRSF2 Exon 1 (44.2%), TET2 Exon 4 (65.2%), and CBL Exons 8 and 9 (36.4% and 6.8%). On November 22, 2024, a lumbar puncture demonstrated cerebrospinal fluid negative for malignant cells, with scattered lymphoid cells, rare monocytes, and erythrocytes, and she concurrently received intrathecal methotrexate and hydrocortisone. Therapeutically, she received high‐dose cytarabine (HiDAC) from November 25–27, 2024.

A bone marrow aspiration and biopsy on December 11, 2024, was inconclusive, revealing marrow replaced by fibrosis with scattered mature lymphocytes and no blasts or hematopoietic elements; flow cytometry was acellular and nondiagnostic. The Day‐17 post‐HiDAC marrow obtained on December 22, 2024, showed a hypoplastic marrow without morphologic evidence of leukemia; however, fine‐needle aspiration of a persistent left upper abdominal wall skin mass remained positive for leukemia. A repeat bone marrow biopsy performed on January 27, 2025, demonstrated a hypercellular marrow with features suggestive of refractory leukemia, with immunostains pending at that time.

**Figure 3:**
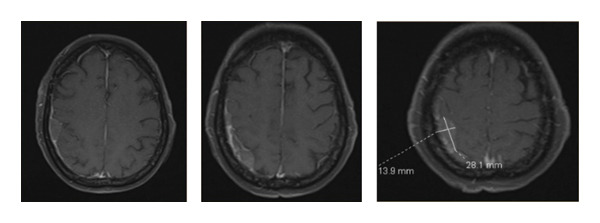
Axial postcontrast T1‐weighted MRI brain obtained one month after intrathecal chemotherapy. MRI demonstrates interval decrease in dural thickening along the frontal and parietal convexities with new nodular dural components along the right parietal and temporal convexities. Background findings include chronic microvascular ischemic disease and multiple cheronic lacunar infarcts.

**Figure 4:**
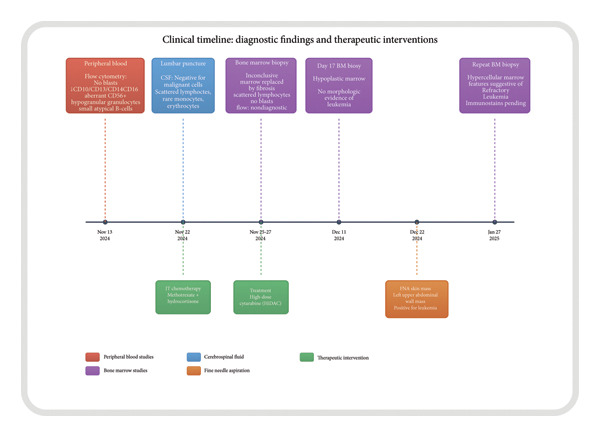
Timeline of diagnostic and therapeutic milestones, from November 2024 to January 2025. Events include peripheral blood flow cytometry, lumbar puncture with intrathecal chemotherapy, systemic HiDAC chemotherapy, serial bone marrow biopsies, fine needle aspiration of skin mass, and final findings of refractory leukemia. Color coding: red = peripheral blood studies, blue = cerebrospinal fluid, purple = bone marrow studies, orange = fine needle aspiration, and green = therapeutic interventions.

Serial brain MRIs on December 9, 2024, and December 16, 2024, demonstrated marked improvement in the previously noted dural lesions, consistent with the treatment response of CNS leukemia (Figure 3). A summary of her therapeutic course and response timeline is illustrated in Figure 4.

On follow‐up, laboratory studies revealed hemoglobin of 8.4 g/dL, white blood cell count of 6.07 × 10^9^/L, hematocrit of 24%, and platelets of 20 × 10^9^/L. Repeated brain MRI demonstrated stable dural thickening along the frontal and parietal convexities without new lesions. Following the intrathecal and systemic chemotherapy, the patient demonstrated improvement in neurological status with resolution of fluctuating confusion.

Importantly, despite the absence of a definitive hematologic diagnosis early in her clinical course, the patient’s clinical and radiographic response to leukemia‐directed therapy, together with the subsequent bone marrow biopsy demonstrating refractory leukemia, supported the ultimate diagnosis.

## 3. Discussion

The progression from myeloid sarcoma to AML with CNS involvement, as observed in our patient, underscores the complex and dynamic nature of myeloid neoplasms. This case highlights several important aspects of disease progression, diagnostic challenges, and therapeutic considerations that merit further discussion.

### 3.1. Disease Progression and Diagnosis

The progression from myeloid sarcoma to AML with CNS involvement, as in our patient, illustrates the continuum and biological plasticity of myeloid neoplasms. Myeloid sarcoma is a rare extramedullary manifestation of myeloid disease, which may present antecedently, concurrently, or in relapse of AML [6]. In series of myeloid sarcoma, overall survival is poor (median of about 4 months), though those who undergo allogeneic stem cell transplantation may achieve better outcomes (median up to 19 months) in selected cases [7]. In the setting of CNS manifestations, intracranial involvement of myeloid sarcoma has been reported, sometimes preceding overt AML diagnosis [8].

### 3.2. Diagnostic Challenges

Diagnosing CNS leukemia in patients with myeloid neoplasms is fraught with difficulty. Neurological symptoms can mimic complications of treatment, metabolic derangements, or vascular events. Thus, a high index of suspicion is required. Evaluations typically include CSF cytology, flow cytometry, and molecular testing, although sensitivity is limited when CSF cellularity is low. Neuroimaging, especially MRI, is an essential adjunct but often yields nonspecific findings, necessitating correlation with clinical and laboratory data [9]. In adult AML cohorts, CNS involvement is uncommon but recognized: for example, in the SAL trials, symptomatic CNS involvement occurred in 0.6% at diagnosis and 2.9% at relapse [10]. In a meta‐analysis, CNS involvement in adults with AML was associated with a hazard ratio of ∼1.34 for worse overall survival compared to those without CNS disease [11].

### 3.3. Treatment Considerations

Managing AML with CNS involvement must balance systemic disease control and intracerebral disease. Standard AML induction/consolidation regimens remain foundational, while CNS‐directed therapies—most commonly intrathecal chemotherapy (e.g., methotrexate and cytarabine) and in some cases high‐dose systemic agents that penetrate the CSF—are required concomitantly [9]. Radiation therapy may be reserved for refractory or bulky disease but carries risks of long‐term neurotoxicity in adults [9]. Allogeneic hematopoietic stem cell transplantation (allo‐HSCT) is often considered in high‐risk AML, particularly for patients with extramedullary or CNS disease, either in first remission or as salvage therapy [12].

In adult AML patients with CNS involvement, intrathecal therapy is often effective in rapid CSF clearance and neurologic symptom improvement. In a multi‐institutional series of 52 adult AML patients with CNS disease, 84% cleared their CSF (often after just one dose of intrathecal therapy) and 69% of those with neurologic symptoms had symptomatic improvement. Median overall survival was 9.3 months when CNS involvement was identified before or during induction and 3.5 months for CNS involvement identified at relapse [13].

### 3.4. Prognostic Implications

CNS involvement in AML has historically been associated with adverse outcomes. In adult cohorts, symptomatic CNS disease at diagnosis is linked to inferior survival: for example, the SAL trial analysis showed 5‐year overall survival of 11% in AML patients with CNS involvement versus ∼30% in those without [10]. The meta‐analysis corroborated this, demonstrating a ∼1.34‐fold increased hazard of death [11]. In the multi‐institutional series, outcomes were heavily stratified by timing of CNS detection (earlier detection correlated with better survival) [13].

In the transplant setting, the presence of CNS disease does not always independently worsen survival post–allo‐HSCT but may be associated with higher relapse risk. In a 2019 analysis, prior CNS disease was not an independent adverse factor for survival after transplantation, though these patients did exhibit higher relapse incidence [14]. In a registry‐based study of CNS relapse post–allo‐HSCT in leukemia, AML CNS relapse incidence was ∼1.8%, and pretransplant CNS involvement was an independent risk for CNS relapse and associated with inferior 3‐year overall survival [15].

Given this landscape, our case is illustrative: despite inconclusive early diagnostic studies, the neurological improvement, CSF clearance, and MRI regression, together with later marrow confirmation, argue strongly for a diagnosis of CNS AML. This underscores the importance of integrating clinical course into the diagnostic algorithm when classic features are lacking.

Our case was also complicated by a social determinants barrier of language. As a Korean immigrant with limited English proficiency, our patient faced significant challenges in accessing and navigating the healthcare system. These language barriers likely contributed to delays in diagnosis and treatment, potentially impacting the disease course and outcome [16]. This case highlights the critical need for improved language support services in healthcare settings, cultural competency training for healthcare providers, patient education materials in multiple languages, and community outreach programs to enhance health literacy among immigrant populations.

## 4. Conclusion

This case report highlights the complex clinical course of a patient with myeloid sarcoma progressing to AML with CNS involvement. It underscores the importance of maintaining a high index of suspicion for disease progression and CNS involvement in patients with myeloid neoplasms. The progression from myeloid sarcoma to AML and subsequent CNS leukemia represents a continuum of disease that requires vigilant monitoring and adaptable treatment strategies. While current management approaches have improved outcomes for some patients, the overall prognosis for those with CNS involvement remains poor.

Advancements in molecular diagnostics, targeted therapies, and immunotherapies offer hope for improved outcomes in the future. However, further research is needed to optimize treatment strategies and develop more effective therapies for this challenging group of patients. As our understanding of the biology of myeloid neoplasms continues to evolve, so too will our ability to provide more personalized and effective care for patients facing these complex hematological malignancies. This case not only underscores the importance of early diagnosis and treatment of CNS leukemia but also highlights the significant impact of language barriers and health disparities on patient outcomes.

## Ethics Statement

This study was conducted in accordance with institutional guidelines and ethical standards.

## Consent

Written informed consent was obtained from the patient (or the patient’s legal representative) for publication of this case report and accompanying images.

## Disclosure

All authors approved the final version and agree to be accountable for all aspects of the work.

## Conflicts of Interest

The authors declare no conflicts of interest.

## Author Contributions

All authors contributed to the conception, drafting, and critical revision of the manuscript.

## Funding

The authors received no specific grant from any funding agency in the public, commercial, or not‐for‐profit sectors.

## Data Availability

All data supporting the findings of this study are contained within the article itself. No supporting information are associated with this submission.

## References

[1] Avni B. and Koren-Michowitz M. , Myeloid Sarcoma: Current Approach and Therapeutic Options, Therapeutic Advances in Hematology. (2011) 2, no. 5, 309–316, 10.1177/2040620711410774, 2-s2.0-84993824965. 23556098 PMC3573418

[2] Deák D. , Teleanu E. , Elekes R. et al., Central Nervous System Involvement in Acute Myeloid Leukemia: A Narrative Review, Annals of Hematology. (2021) 100, no. 3, 635–649, 10.1007/s00277-020-04304-9.33216196

[3] Suárez E. U. , Castaño-Bonilla T. , Salgado R. et al., Therapeutics of Acute Myeloid Leukemia With Central Nervous System Involvement, Clinical Hematology International. (2025) 7, no. 1, 40–46, 10.46989/001c.131722. 40083897 PMC11906164

[4] Blum S. , Shigematsu A. , Takahashi S. et al., Incidence and Outcome of Central Nervous System Relapse in Acute Myeloid Leukemia: A Nationwide Retrospective Analysis, Haematologica. (2024) 109, no. 2, 427–436, 10.3324/haematol.2023.284858.

[5] Bar M. , Tong W. , Othus M. et al., Central Nervous System Involvement in Acute Myeloid Leukemia Patients Undergoing Hematopoietic Cell Transplantation, Biology of Blood and Marrow Transplantation. (2015) 21, no. 3, 546–551, 10.1016/j.bbmt.2014.11.683, 2-s2.0-84922826707. 25545726 PMC4720268

[6] Pileri S. A. , Ascani S. , Cox M. C. et al., Myeloid Sarcoma: Clinico-Pathologic, Phenotypic and Cytogenetic Analysis of 92 Adult Patients, Leukemia. (2007) 21, no. 2, 340–350, 10.1038/sj.leu.2404491, 2-s2.0-33846524299. 17170724

[7] Quesada A. E. , Estrov Z. , Champlin R. E. et al., Clinicopathologic Correlates and Prognostic Significance of Myeloid Sarcoma in Adults: A 10-Year Single-Institution Experience, Scientific Reports. (2022) 12, no. 1.

[8] Kawamoto K. , Miyoshi H. , Yoshida N. et al., Clinicopathological, Cytogenetic, and Prognostic Analysis of 131 Myeloid Sarcoma Patients, The American Journal of Surgical Pathology. (2016) 40, no. 11, 1473–1483, 10.1097/pas.0000000000000727, 2-s2.0-84987892171. 27631510

[9] Alakel N. , Moritz J. , Schetelig J. , and Bornhäuser M. , Central Nervous System Involvement in Acute Myeloid Leukemia, Haematologica. (2022) 107, no. 1, 22–32.

[10] Kurosawa S. , Yamauchi T. , Sakura T. et al., Prognosis of Patients With Acute Myeloid Leukemia Who Developed Central Nervous System Involvement: A Multicenter Retrospective Study of the Japan Adult Leukemia Study Group, Annals of Hematology. (2017) 96, no. 1, 99–106.27699447

[11] Wang R. , Huang Y. , Sun C. et al., Prognostic Impact of Central Nervous System Involvement in Adults With Acute Myeloid Leukemia: A Systematic Review and Meta-Analysis, Frontiers in Oncology. (2024) 14.

[12] Zhao X.-S. , Liu K.-Y. , Xu Li-P. et al., Impact of Central Nervous System Involvement at Diagnosis on Allogeneic Hematopoietic Stem Cell Transplantation Outcomes in Acute Myeloid Leukemia, Bone Marrow Transplantation. (2019) 54, no. 12, 1964–1971.

[13] Rosenfield C. , Khot A. , Perry A. M. et al., Outcomes of Central Nervous System Involvement in Adults With Acute Myeloid Leukemia: A Multicenter, Retrospective Analysis, Leukemia and Lymphoma. (2023) 64, no. 11, 1101–1108.

[14] Orvain C. , Chevallier P. , Clavert A. et al., CNS Involvement in Acute Myeloid Leukemia at Diagnosis and Relapse: Prognostic Impact and Outcomes After Allogeneic Transplantation, Bone Marrow Transplantation. (2019) 54, no. 12, 1972–1979.

[15] Blum S. , Chalandon Y. , Labopin M. et al., Incidence and Outcome of Central Nervous System Relapse After Hematopoietic Stem Cell Transplantation in Patients Suffering From Acute Myeloid Leukemia and Acute Lymphoblastic Leukemia: A Study From the Acute Leukemia Working Party of the European Society for Blood and Marrow Transplantation, Haematologica. (2024) 109, no. 7, 2346–2350, 10.3324/haematol.2023.284858. 38450525 PMC11215349

[16] Pandey M. , Maina R. G. , Amoyaw J. et al., Impacts of English Language Proficiency on Healthcare Access, Use, and Outcomes Among Immigrants: A Qualitative Study, BMC Health Services Research. (2021) 21, no. 1, 10.1186/s12913-021-06750-4. PMC8314461 34311712

